# A Postmortem Study on Ultrasonographic and Computed Tomographic Measurements of the Concavity of the Solar Surface in the Distal Phalanx of Cow Hindlimb Claws

**DOI:** 10.3390/ani16050812

**Published:** 2026-03-05

**Authors:** Katsufumi Oshita, Takeshi Tsuka, Ryo Nishimura

**Affiliations:** 1 Oshita Large Animal Clinic, 701-2, Imaida, Kabe, Asakita-ku, Hiroshima 7310234, Japan; oochan@krf.biglobe.ne.jp; 2 Clinical Veterinary Sciences, Joint Department of Veterinary Medicine, Faculty of Agriculture, Tottori University, 4-101, Koyama-Minami, Tottori 6808553, Japan; ryon@tottori-u.ac.jp; 3 Joint Graduate School of Veterinary Sciences, Tottori University, 4-101, Koyama-Minami, Tottori 6808553, Japan

**Keywords:** bovine, morphometry, new bone formation, tuberculum flexorium, ultrasonography

## Abstract

New bone formations (NBFs) in the tuberculum flexorium of the distal phalanx (DP) are considered one of the etiological factors inducing claw horn disruption lesions, which frequently occur in the lateral claws of cow hindlimbs. The pathological bone changes in the DP are often not clearly visible using routine ultrasonography (US) of bovine claws. Thus, to enable an evaluation of these changes using US, this report investigated the utility of the angle in the concavity of the solar surface of the DP (referred to as the DP-SS angle), which varies with the severity of NBFs in the tuberculum flexorium. A total of 484 pairs of hindlimbs from 242 lactating Holstein cows collected at a slaughterhouse were examined using computed tomography (CT), and 274 hindlimb pairs from 137 animals were examined using both US and CT. For lateral claws, the DP-SS angle values showed a moderate to strong negative correlation with the NBF width (r = −0.71), while a weak correlation was found for the medial claws (r = −0.25). The DP-SS angles measured using US were significantly (*p* < 0.01) correlated with those measured using CT. Lateral claws with claw diseases had a significantly (*p* < 0.01) lower value (158.6°) than claws without claw diseases (161.6°). The value could not be measured using US for 51.4% and 30.0% of claws with double soles or a sole ulcer/sole crack, respectively, because the DP echotextures were unclear in the US. In the CT analyses, the DP-SS angle was the lowest in lateral claws with a sole ulcer/sole crack (154.4°); this value was significantly smaller (*p* < 0.01) than that of lateral claws with white line disease (162.3°) or a thin sole/thin sole toe ulcer (163.5°), as well as that of lateral claws without claw diseases (161.6°). In the US analyses, the value for lateral claws with a sole ulcer/sole crack (156.7°) tended to be slightly larger compared with the CT measurements, although it was significantly different from the value for claws without claw diseases (163.0°). The DP-SS angle is considered accurate for evaluating the degree of an NBF in the lateral claws of cow hindlimbs. The preliminary standard value of 160° was considered based on the CT analyses. However, the normal and abnormal thresholds of this value should be confirmed through the further field use of claw US because of the difference between the US and CT measurements. Additionally, creating disease-specific thresholds may be required owing to the considerable variation.

## Simple Summary

When using a portable ultrasound machine on bovine claws, the image quality is often poor. It is difficult to clearly visualize the solar surface of the distal phalanx (DP) on claw ultrasonograms. This makes it challenging to evaluate the varying severities of new bone formations, which frequently occur downward in the area of the tuberculum flexorium. This study proposes the use of the angle in the concavity of the solar surface of the DP, referred to as the DP-SS angle, as a parameter to indirectly evaluate the degree of new bone formations. The ultrasonographic measurement method was designed so that two ultrasonographic images obtained when scanning the dorsal and plantar regions of the sole surface separately are used to calculate the DP-SS angle. In ultrasonographic analyses of 274 hindlimb pairs from 137 Holstein cows from a slaughterhouse, the average values for diseased claws, except for those with interdigital dermatitis or white line disease, were below the standard value of 160° established preliminarily based on the ranges of the values for claws without claw diseases. In particular, claws with sole ulcers/sole cracks had an average value of 156.7°, which was significantly smaller than the values for claws without claw diseases. This ultrasonographic protocol using the DP-SS angle seems useful for evaluating the pathogenic role of new bone formations and estimating therapeutic effects and prognoses in the field of claw ultrasonography.

## 1. Introduction

Claw horn disruption lesions are considered to occur due to damage to the germinal epithelium, resulting in a poor-quality sole horn [1]. Claw horn disruption lesions include sole ulcers, sole hemorrhages, and white line disease, which mostly occur in the lateral claw of a cow’s hindlimb [2,3]. Sole ulcers can develop from a sole hemorrhage during the pathological process of the sole horn and are associated with the failure of horn production [1,3]. Weakened parts of the white line are easily separated, even under normal weight-bearing forces [4]. Various factors contribute to the occurrence of claw horn disruption lesions, including a cow’s age and parity, lactation periods, and insufficient nutrition, which are associated with changes in the fat quality and the thickness of the digital cushion [1,5]. The pathogenesis of the lesions also includes mechanical compression from the distal phalanx (DP), which damages underlying soft tissues such as the corium and sole horn [2,3,6,7]. A new bone formation (NBF) in the DP is considered another cause of mechanical damage [2,6,7,8]. Previous histological studies have shown that most pathological bone changes in the DP involve bone-modeling lesions [2]. The new bones tend to develop downward in proportion to their horizontal extensions, from the abaxial to axial region of the tuberculum flexorium [2,7]. Additionally, a larger NBF in the lateral claws of the hindlimbs, compared with the medial claws, is a hypothetical cause for the claw horn disruption lesions that typically occur in this region [2,7].

Computed tomography (CT) has previously been used for basic etiological assessments of claw horn disruption lesions using slaughterhouse specimens [2,7,9,10]. CT is superior for imaging the structure-associated differences in attenuating X-ray beams, which allows for a distinction to be made between the existing bone and new bone. CT has considerable value for evaluating NBFs in the DP, as well as the thickness of the sole horn and soft tissue layers, particularly when using reconstruction functions to generate multi-directional, two-dimensional, and three-dimensional CT images [2,7]. However, CT is not clinically applicable for identifying NBFs as a causative factor in claw horn disruption in living cows.

Ultrasonography (US) is a useful imaging tool for examining the interior of a bovine claw when applied from the basal surface of the sole horn. The basal structure of the bovine claw is represented by three echogenic layers: the echogenic sole horn; the more hypoechoic underlying soft tissue layers, including the corium; and the hyperechoic line of the solar DP surface, visualized at depth [9,11]. US helps measure the sole horn thickness [9,11,12,13,14,15], the thickness of the soft tissue layers or digital cushion [15,16,17,18,19,20,21], and the distance between the sole surface and the solar DP surface [14]. However, US has rarely been used to diagnose bone lesions of the DP within bovine claws [22]. This is because the inferior function of US to clearly represent bone structures commonly makes it difficult to distinguish the echotexture of NBFs from that of the solar DP surface [11,14]. Thus, US is not applicable in quantitative analyses to measure the magnitudes (such as the width and length) of pathological osseous changes in the DP. It is necessary for US analyses to develop an alternative to the direct measurement method used in CT analyses [2,7] when evaluating the etiological role of DP NBFs in the induction of claw horn disruption lesions. During our clinical use of a portable US machine on diseased claws, we observed changes in the bone echotexture at the tuberculum flexorium area that affected the angle in the concavity of the solar surface (SS) of the DP, referred to as the DP-SS angle. Dependent on the NBF levels, new bones rising steeply from the solar surface of the tuberculum flexorium can lead to smaller (more acute) concavity angles of the DP compared with the original angles of the solar DP surface. Thus, US measurements of the DP-SS angle are considered a useful indirect indicator of NBFs in the tuberculum flexorium. Internal structures of the bovine claw are commonly observed in dorsal and plantar US images through a small field of view in a portable US machine when scanning from the sole horn [11,23]. In the present report, the DP-SS angle was calculated using a common scanning technique, by summing the two values obtained from scanning to divide the bone echotexture into dorsal and plantar parts.

The present report was designed to evaluate the measurement accuracy of bovine claw US using CT values by comparing imaging data obtained from the same bovine claws when examined with US and CT postmortem. Quantitative analyses of the bovine claw CT were used to identify (1) the consistency of the DP-SS angle, calculated by summing two angles; (2) the correlation between the DP-SS angle and the NBF width; and (3) a comparison between the values in claws with or without claw diseases. The US measurements of the DP-SS angle were investigated for (1) the measurement accuracy, as determined by evaluating the consistency with the CT value for the same claws, and (2) the detection sensitivity for claw diseases, followed by estimating the abnormal value. Additionally, these results were discussed from the point of view of (1) the technical meaningfulness of the DP-SS angle in bovine claw US, (2) the utility of the DP-SS angle for differentiating between claws with and without claw diseases, (3) the limitations on the utilization of this technique in the field, and (4) the study limitations.

## 2. Materials and Methods

### 2.1. Specimens

The specimens were 242 pairs of hindlimbs from 242 lactating Holstein-Friesian cows (#1 to #242) that were obtained from a slaughterhouse (Tottori Meat Inspection Center, Tottori Prefectural Government, Tottori, Japan) between August 2006 and March 2019. A total of 236 of the 242 animals were aged between 23 and 144 months at the time of culling, whereas the ages of the remaining six animals were unknown. Of the 242 animals, 108 were housed in free-stall or free-barn units and 129 were housed in tie-stall barns. The housing type was unknown for the remaining five cows. The reasons for culling included orthopedic diseases (such as hip dislocation or claw diseases; n = 69), mastitis (n = 33), gastrointestinal diseases (e.g., displaced abomasum or enteritis; n = 23), metabolic diseases (e.g., hypocalcemia; n = 16), infectious diseases (e.g., peritonitis and pneumonia; n = 10), neurological diseases (n = 5), low productivity (e.g., poor milk production or reproductive performance; n = 8), and unknown reasons (n = 78). Based on macroscopic observations of light-trimmed sole surfaces or cut surfaces of the slaughterhouse specimens (n = 968; 484 medial and 484 lateral claws), the claws were divided into two groups: claws in which claw diseases were detected were grouped as CD (+) (n = 245; 86 medial and 159 lateral claws), and claws in which no claw diseases were detected were grouped as CD (−) (n = 723; 398 medial and 325 lateral claws). In the CD (+) group, the claw diseases were categorized into six types: double soles (n = 64; 28 medial and 36 lateral claws); white line disease (n = 53; 18 medial and 35 lateral claws); sole ulcers/sole cracks (n = 50; 3 medial and 47 lateral claws); thin sole/thin sole toe ulcers (n = 37; 21 medial and 16 lateral claws); interdigital dermatitis (n = 17; 10 medial and 7 lateral claws); and other diseases, including various laminitic changes such as sole discoloration and deformed hooves (n = 24; 6 medial and 18 lateral claws). All specimens were examined with the imaging modalities within six hours after collecting them from a slaughterhouse.

### 2.2. US Imaging Procedures

**Figure 1:**
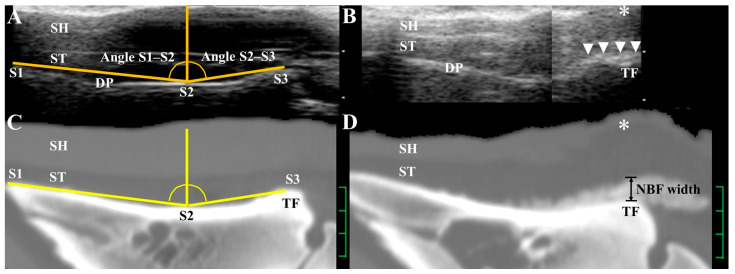
Mid-sagittal US and 2-dimensional CT images used to measure the DP-SS angle in the lateral claw of the right hindlimb in an 86-month-old Holstein cow ((**A**) and (**C**), respectively; DP-SS angle = 166.2°), and the lateral claw of the right hindlimb in a 70-month-old Holstein cow ((**B**) and (**D**), respectively; DP-SS angle = 152.7°). (**A**) On the US image, the angle was measured between two virtual lines: one passing through points S1 and S2, and another drawn vertically from point S2 to the solar DP surface. This was defined as angle S1–S2. Angle S2–S3 was measured between the vertical line at point S2 and the line connecting points S2 and S3. (**B**) Thickening in the DP line (arrowheads) was evident at the area of the tuberculum flexorium (TF), corresponding with the protruded part. A partial defect in the sole horn (SH) (asterisk) was also seen on the US. (**C**) On the CT image, the DP-SS angle was measured by the angle formed by the virtual line passing through points S1, S2, and S3. The intersection center of the angle is located on point S2. (**D**) CT identified an NBF at the area of the TF. The NBF width was measured using the maximum width between the hyperattenuating bone line and the new bone’s margin. Thinning in the SH (asterisk) was seen near the NBF lesion, corresponding to the thickened soft tissue layers (ST). Scale = 10 mm on US image, and 5 mm on CT image.

A portable US machine (HONDA HS-101V; Honda Electronics Co., Ltd., Toyohashi, Aichi, Japan) was used to perform a postmortem examination of 274 medial and lateral claws from 137 cows (#106 to #242) obtained from the slaughterhouse between July 2012 and March 2019. Of the total 548 examined claws, 35 had a double sole, 4 had white line disease, 30 had a sole ulcer/sole crack, 5 had a thin sole/thin sole toe ulcer, 4 had interdigital dermatitis, and 9 had other claw diseases. The remaining 461 claws were grouped into CD (−). A 5 MHz linear transducer was applied to the sole surfaces of the claws, which were prepared with light trimming to create a smooth, flat surface while maintaining the original claw shape. Transmission gel was applied to the contact surface of the transducer. The scanning site ranged from the midpoint of the toe area to the middle of the heel area, with the transducer applied along the dorsal–plantar axis [9]. The longitudinal US plane displayed three echogenic lines corresponding to the surface of the sole horn, the border between the sole horn and the soft tissue layers, and the solar DP surface (Figure 1A,B) [11]. In the present study, the claws were scanned in two parts: a dorsal claw US displayed the anatomical level between point S1 (the most apex margin of the DP) and point S2 (the deepest concavity of the DP), and a plantar claw US showed the level between points S2 and S3 (the tuberculum flexorium, including the insertion of the deep digital flexor tendon) [9].

### 2.3. CT Imaging Procedures

A slip-ring scanner (Pronto SE, Hitachi, Tokyo, Japan) with X-ray tube settings of 100–120 kVp, 100 mA, and a 2.5 mm slice thickness (used between April 2006 and July 2011) and a 16-section multidetector scanner (ECLOS, Hitachi, Tokyo, Japan) with X-ray tube settings of 120 kVp, 175 mA, and a 0.625 mm slice thickness (used between July 2011 and March 2019) were used for animals #1 to #97 and #98 to #242, respectively. Sagittal two-dimensional CT images were obtained using an image analysis system (AZE Virtual Place, AZE Corp., Tokyo, Japan) corresponding to the typical US scanning section of bovine claws [9,11]. On basic sagittal CT images, the solar DP surface appeared as a smooth line of bone density, clearly distinguishable from the densities of the sole horn and soft tissue layers. The border between these layers was defined by differing attenuation values; the CT density of the sole horn was generally higher than that of the soft tissue layers. On CTs, the irregularity and thickening of the bone cortex commonly represented an NBF on the solar DP surface. The CT densities of the NBF parts were lower than those of the bone line, revealing a clear border between the solar DP line and new bone structures on the CT.

### 2.4. Quantitative Evaluations of CT and US Images

On the US images of 274 medial and lateral claws (#106 to #242), the DP-SS angle was measured using image analysis software (ImageJ 1.53a, NIH, Bethesda, MD, USA) by one author (K.O.). The DP-SS angle was measured in the following three steps: (1) For the dorsal claw US, two virtual lines were drawn: one between points S1 and S2, and one vertical to the solar DP surface at point S2 (Figure 1A,C). The angle between these lines was recorded as angle S1–S2. (2) For the plantar claw US, angle S2–S3 was measured between the vertical line at point S2 and a line connecting points S2 and S3. (3) The DP-SS angle was calculated as the sum of angles S1–S2 and S2–S3. Thus, the intersection center of the two virtual lines was located on point S2.

One author (T.T.) performed the CT measurements. On the CT, angles S1–S2 and S2–S3 were measured in the same manner as in the US measurements for 210 claws from 105 animals (#1 to #105). Additionally, for all the animals (#1 to #242), the DP-SS angle was measured in the following two steps: (1) A virtual line was drawn connecting points S1, S2, and S3, the intersection center of which was located on point S2 (Figure 1C). (2) The value was obtained by measuring the angle made by the virtual line at point S2. The NBF widths were measured by observing the maximum vertical (downward) protrusion of new bones from the hyperattenuating bone lines at the level of the tuberculum flexorium on the CT [2,7] (Figure 1D). In the measurement of NBF widths, the CT images were magnified approximately 5 times. For both US and CT, each measurement was recorded as the average of three replicates.

### 2.5. Statistical Analysis

#### 2.5.1. Comparison of Two Different Evaluations of DP-SS Angle Using CT

Using the CTs of 210 medial and lateral claws (#1 to #105), two types of DP-SS angles were analyzed: the value calculated by adding angles S1–S2 and S2–S3 (referred to as the CT-calculated DP-SS angle) and the value measured as the angle formed by the line connecting points S1, S2, and S3 (referred to as the CT-measured DP-SS angle). Angle S1–S2, angle S2–S3, and the CT-calculated DP-SS angle were statistically compared between the medial, lateral, and total claws using a one-way analysis of variance (ANOVA). Differences between the CT-calculated and CT-measured DP-SS angles were assessed for the medial, lateral, and total groups using Student’s t-test. The concordance correlation coefficient (CCC) was used to assess the statistical relationships between the CT-calculated and CT-measured DP-SS angles for the medial claws (n = 210), lateral claws (n = 210), and total claws (n = 420).

#### 2.5.2. Comparison Between NBF Widths and DP-SS Angles Using CT

Using the CT measurement values for all the specimens (#1 to #242), statistical relationships between the NBF widths and the DP-SS angles were assessed using the Pearson correlation coefficients (r) and 95% confidence intervals (CIs) for the medial claws (n = 484), lateral claws (n = 484), and total claws (n = 968).

#### 2.5.3. Association of Claw Diseases with DP-SS Angles and NBF Widths

The CT-measured DP-SS angles and NBF widths were statistically analyzed between the medial (n = 484), lateral (n = 484), and total claws (n = 968) of the specimens (#1 to #242) using an ANOVA test. An ANOVA was also used to make statistical comparisons between the medial, lateral, and total claws in each CD (−) and CD (+) group. Furthermore, an ANOVA was applied to compare the CD (−), CD (+), and total claws in each of the medial, lateral, and total claw groups. The DP-SS angles and NBF widths of the six types of claw diseases were statistically compared with those of the CD (−) group using an ANOVA. In the present report, the proportions of claws with DP-SS angles < 150°, 150–<160°, and ≥160° were calculated for each of the six claw disease types and the CD (−) group. Differences in the proportions between these seven groups were compared using a 2 × 7 chi-square test for the 150–<160° and ≥160° categories and Fisher’s exact test for the <150° category in each of the medial, lateral, and total claw groups.

#### 2.5.4. Comparison Between DP-SS Angles Measured Using US and CT

This analysis was performed using 274 medial and lateral claws of 137 cows (#106 to #242) that were examined using both US and CT. The statistical relationships between the DP-SS angles obtained from US (by summing angles S1–S2 and S2–S3) and from CT (CT-measured DP-SS angles) were assessed using the CCC for the medial claws (n = 274), lateral claws (n = 274), and total claws (n = 548). Mean relative errors were also calculated for the medial, lateral, and total claws using the following formula: (CT-measured value − US-measured value)/CT-measured value × 100 (%). The rates of US-examined claws for which DP-SS angles could be measured were determined for each of the six claw disease types and the CD (−) group. The rates in each disease group were compared with that of the CD (−) group using Fisher’s exact test. The DP-SS angles were also compared between the six disease types and CD (−) using an ANOVA.

All the data were analyzed as the mean ± standard deviation (SD). The Bonferroni test was used for post hoc comparisons in all ANOVA analyses throughout the study. A *p*-value of <0.01 was considered statistically significant.

## 3. Results

### 3.1. Comparison of Two Different Evaluations of DP-SS Angle Using CT

For angle S1–S2, there was no significant difference between the medial, lateral, and total claw groups (Table 1). However, angle S2–S3 in the lateral claws was significantly (*p* < 0.01) smaller than those in the medial and total claw groups. The lateral claw group also had a significantly (*p* < 0.01) smaller CT-calculated DP-SS angle than the medial and total claw groups. There were no significant differences between the CT-calculated and CT-measured DP-SS angles in the medial, lateral, and total claws. The CCCs (95% CI) between the CT-calculated and CT-measured DP-SS angles were 0.93 (0.92 to 0.94), 0.97 (0.97 to 0.98), and 0.96 (0.96 to 0.97) in the medial, lateral, and total claw groups, respectively.

### 3.2. Comparison Between NBF Widths and DP-SS Angles Using CT

**Figure 2:**
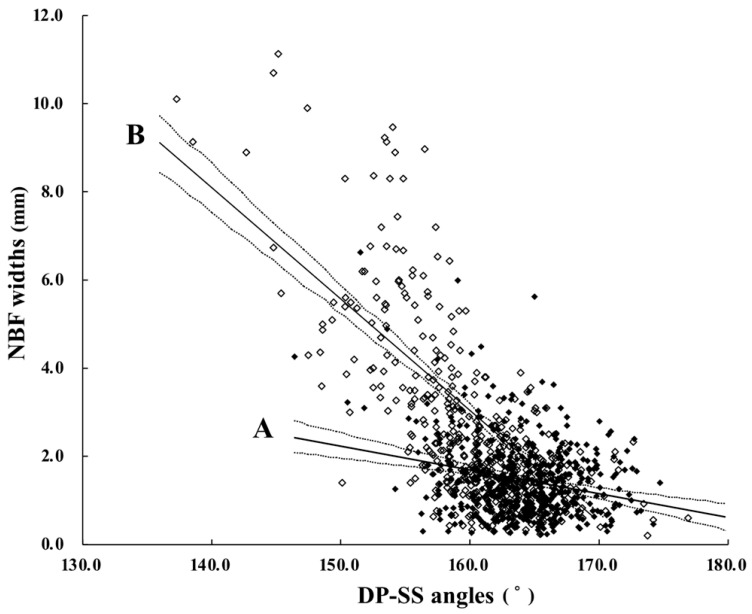
Correlations between DP-SS angles and NBF widths measured using CT in (A) medial and (B) lateral claws. Closed and open diamonds represent measured values in medial and lateral claws, respectively. Black solid lines indicate the regression line, and black broken lines indicate the 95% confidence interval. Values for medial claws show a wider distribution around the regression line compared with those for lateral claws.

On the CT images, the average DP-SS angles (CT-measured) exceeded 160° for the medial, lateral, and total claw groups in the CD (−) group and for all claws (Table 2). The value for the lateral claws was significantly (*p* < 0.01) smaller than those for the medial and total claws. The NBF width in the lateral claw group was significantly (*p* < 0.01) greater than in the medial and total claw groups (Table 3). Negative correlations were found between the DP-SS angles and the NBF width of both the medial and lateral claw groups (Figure 2 (A,B)). The Pearson correlation coefficients were weak (r = −0.25, *p* < 0.01) for medial claws, moderate to strong (r = −0.71, *p* < 0.01) for lateral claws, and moderate overall (r = −0.62, *p* < 0.01).

### 3.3. Association of Claw Diseases with DP-SS Angles and NBF Widths

The DP-SS angles of the medial, lateral, and total claw groups were significantly (*p* < 0.01) different from each other in both the CD (−) and total groups (Table 2). A similar pattern was observed for the NBF widths in the CD (−) and total groups (Table 3). In the CD (+) group, significant (*p* < 0.01) differences in the DP-SS angles and NBF widths were found for the medial claws compared with those for the lateral and total affected claws. When comparing between the CD (−), CD (+), and total groups, the DP-SS angles of the lateral and total claws were significantly (*p* < 0.01) smaller in the CD (+) group than in the CD (−) and total groups, while no significant differences were observed between these groups for the medial claws. The NBF widths significantly differed between the CD (−), CD (+), and total groups for the lateral and total claws. Additionally, the NBF width was significantly (*p* < 0.01) different between the CD (−) and CD (+) groups. Lateral claws affected by a sole ulcer/sole crack had significantly (*p* < 0.01) smaller angles than the medial affected claws (Table 2), while no significant differences were found in the NBF widths (Table 3). Among the medial claws, neither the DP-SS angles nor the NBF widths differed significantly between the six claw disease types and the CD (−) group. For the lateral claws, the DP-SS angles and NBF widths in claws with a sole ulcer/sole crack were significantly (*p* < 0.01) smaller than those in claws with white line disease, claws with a thin sole/thin sole toe ulcer, and the CD (−) group. For the total claws, both the DP-SS angles and the NBF widths in the sole ulcer/sole crack group differed significantly (*p* < 0.01) from those of the other five claw disease types and the CD (−) group. DP-SS angles < 150° were measured in 0.6% and 0.3% of the lateral and total claws in the CD (−) group, respectively. These values were significantly (*p* < 0.01) lower than the rates for claws with a double sole, a sole ulcer/sole crack, or other claw diseases (Table 4). In lateral and total claws with white line disease, the rates of a DP-SS angle < 150° were significantly (*p* < 0.01) lower than in those with a sole ulcer/sole crack. Regarding DP-SS angles 150–<160°, the rate in the sole ulcer/sole crack group was significantly (*p* < 0.01) higher than in the double sole, white line disease, and thin sole/thin sole toe ulcer groups for lateral claws, and higher than in all other five claw disease groups and the CD (−) group for the total claws.

### 3.4. Comparison Between DP-SS Angles Measured Using US and CT

The CCCs (95% CI) between the DP-SS angles measured via US and CT were 0.85 (0.82 to 0.87), 0.90 (0.89 to 0.92), and 0.89 (0.88 to 0.91) in the medial, lateral, and total claw groups, respectively. The mean (lowest to highest values) relative errors were −0.28% (−5.12% to 3.71%), −0.23% (−4.00% to 4.50%), and −0.25% (−5.12% to 4.50%) for the medial, lateral, and total claw groups, respectively. The DP-SS angles could not be measured using US in 48.6% of the claws with a double sole and 70.0% of those with a sole ulcer/sole crack (Table 5), with significantly (*p* < 0.01) lower measurement rates compared with the CD (−) group. Among the claws where the DP-SS angles could be measured on US owing to identifiable internal structures, the angle in claws with a sole ulcer/sole crack was significantly (*p* < 0.01) smaller than in CD (−), while no significant difference was found for the other five types of claw diseases compared with CD (−).

## 4. Discussion

### 4.1. The Technical Meaningfulness of the DP-SS Angle in Bovine Claw US

US commonly has a poorer capacity to depict bone structures in minute detail compared with CT, which has previously been utilized for measuring bovine claws [2,11]. In human medicine, an improved image quality enables the clear visualization of very small bone lesions along irregular, thickened bone lines [24,25]. An adequate image quality can be achieved using very high US frequencies, ranging from 10 to 18 MHz [24,26]. However, even with high-frequency US transducers, bone lesions can be too small to be distinct from the age-related osseous changes typically observed in healthy individuals [25,26,27]. Additionally, individual variation in the bone echotexture may result in the misidentification of enthesitis in healthy persons [26,27]. In the US scanning of equine hooves, the bone lesions associated with enthesophytes or osteolysis were also represented as irregularities in the solar DP echotexture, accompanied by the thickening of the deep digital flexor tendon [28,29]. Additionally, the normal solar DP surface appears as a slightly irregular hyperechoic line when scanned with 5 to 6 MHz transducers, and as a smooth line when using 7 to 10 MHz transducers [30]. Thus, a higher-frequency transducer, potentially above 7.0 MHz, is required to evaluate irregular bone lesions effectively [28,29,30]. The US frequencies previously used for scanning the sole surface of bovine claws include 5.0 MHz [3,11,14,22,31], 6.0 MHz [17,18], 6.5 MHz [11], 7.0 MHz [13], 7.5 MHz [9,12,19,21], 8.0 MHz [16], and 10.0 MHz [20]. The use of lower-frequency transducers is commonly recommended to clearly visualize the solar DP surface on a bovine claw US, as the sole horn acts as a barrier to the US beam [9,11,12,14,22,32]. However, the bone cortex tends to appear as inconsistently thick and poorly defined in contour when scanned using lower-frequency transducers in bovine claw US [9,11,13,16] (Figure 1B). Such a rough, blurred bone echotexture can hinder the detection of small irregularities along the bone surface [22,31]. Additionally, it remains questionable whether portable US machines can reliably detect small bone irregularities in bovine claws, although they are predominantly used for bovine claw examinations owing to their practical applicability [9,11,12,13,14,20,22,31]. Thus, the DP-SS angle was designed as a quantitative analysis of NBFs that can be used in US evaluations. In the present study, CT analyses using slaughterhouse specimens demonstrated a high measurement accuracy for this value, showing a strong correlation with the NBF width. However, US measurements showed slightly lower CCCs compared with CT, indicating a slightly reduced precision. Additionally, US measurements of the DP-SS angle were 0.28% and 0.23% lower than the values measured using CT for medial and lateral claws, respectively. The reference point S2, as proposed by Kofler et al., was employed to measure the DP-SS angle [9]. The complete alignment of this point across the dorsal and plantar US images was challenging. The DP-SS angle can help evaluate NBFs in the DP during the US scanning of bovine claws, although there is a slight decrease in the measurement accuracy.

CT can clearly distinguish between the initial and terminal phases of an NBF in the DP that are associated with sole ulceration, including pathological bone changes following sole horn perforation [7]. Conversely, US lacks the resolution necessary to differentiate these phases effectively, particularly when using lower-frequency transducers [11]. The NBF severity tends to increase during the transition from the initial to terminal stages [7], and therefore, the DP-SS angle may serve as a useful indicator of the NBF phase.

### 4.2. The Utility of the DP-SS Angle for Differentiating Between Claws with and Without Claw Diseases

The present study indicated that the sensitivity of US measurements for evaluating an NBF tend to be lower in medial claws due to their narrower NBF width range (0.2–6.6 mm) compared with lateral claws (0.2–11.1 mm), as shown in Figure 2. In the medial claws, the correlation coefficient between the DP-SS angle and the NBF width may have decreased slightly because random variation became more noticeable. The random variation seemed to be related to greater variation in the DP size and shape between the bovine claws [33]. Natural variation in DP distortion may also influence the degree of bone protrusion near the tuberculum flexorium [33].

Previous CT measurements of the NBF width showed average values of 2.5 mm in healthy claws, resembling the values in the CD (−) group [11]. Another report revealed widths of approximately 0.5 mm and 0.8 mm in medial and lateral healthy claws, respectively [2]. Additionally, values > 5 mm were measured in approximately 4% of the claws that had previously been examined with CT [2]. On the other hand, the average value was approximately 7.0 mm in ulcerated claws [11]. Unfortunately, the clinical implications of the estimated normal or abnormal values are not fully clarified. The thickness of the soft tissue layers averaged 3.7 to 9.8 mm when using US to examine healthy claws [1,16,18,21]. The CT analysis for medial and lateral healthy claws revealed average values of 6.6 mm and 7.3 mm, respectively [7]. Thus, there was less difference between the NBF width and the soft tissue layer thickness in terms of millimeters. Even if there are millimeter-level variations in the NBF width, bone lesions can result in the relative narrowing of the anatomical space between the solar DP surface and the inner surface of the sole horn, leading to a decrease in the thickness of the soft tissue layers [7].

Although normal and abnormal values for the DP-SS angle are unknown because there are no previous comparable data, a DP-SS angle of 160° was considered the standard reference after a comparison between the measurements for bovine claws with and without claw diseases in Table 2. However, this suggestion is based on the preliminary findings of basic research. Thus, further clinical evaluations of the affected claws using this value in the field will be required to improve this exploratory suggestion. On the other hand, since the angle values differed significantly between medial and lateral claws, it may be more appropriate to establish separate standards for each. This is suggested by the great difference between the medial and lateral claws in the relationship between the NDF width and the DP-SS angle in Figure 2.

In the CT analysis, the preliminary standard value of 160° was more sensitive for evaluating lateral claws with a sole ulcer/sole crack or a double sole; approximately 90% and 60% of these cases had values of <160°, respectively. Furthermore, values close to 155° or 158° might be used as specific thresholds for evaluating claws with a sole ulcer/sole crack or a double sole, respectively, corresponding to an NBF width ranging from 4 to 5 mm based on the graph of lateral claws in Figure 2. An extended NBF at the tuberculum flexorium is believed to contribute to mechanical damage of the corium, resulting in a poor-quality sole horn [2,3,7,10]. Interestingly, the DP-SS angle in claws with white line disease was nearly equivalent to that in non-diseased claws. White line disease may result from the mechanical compression of the germinal epithelium by bone changes, particularly in the abaxial regions of the tuberculum flexorium, as part of the initial phase of an NBF [2,7]. Various factors contribute to the process of white line separation following the weakening of the junction between the sole and wall horns induced by mechanical stress. These factors can include wet or dirty floors and uncomfortable or small stalls, which cause continuous standing [4]. Thus, these factors should be given more consideration in etiological and diagnostic evaluations using the DP-SS angle. An NBF in the abaxial region of the DP cannot be fully evaluated for its association with white line disease using US measurements of the value corresponding to Kofler’s method, as it scans the midline of the sole surface [9]. The use of the claw US method to scan the abaxial side of the sole surface along the white line may be more suitable for claws with white line disease [13].

### 4.3. The Limitations on the Utilization of US Measurements in the Field

In the present report, the US measurements of the DP-SS angle tended to be higher than the CT measurements when examining the same claws. Consequently, a significant difference with the CD (−) group was found only for the sole ulcer group. This depended on the lower measurement rates, as the solar DP surface could not be visualized, especially when scanning the claws with a sole ulcer/sole crack or a double sole. This limitation was primarily attributed to the partially or entirely unclear echotexture of the solar DP surface on the plantar claw US for the ulcerated claws. In such cases, extended defects of the sole horn likely interfered with the penetration of the US beam. When scanning claws with a severe double sole, the wide separation of the affected sole horn appeared to serve as an obstacle that attenuated the US beam. Therefore, improving the ability to measure the DP-SS angle in claws at progressed or chronic stages, where extended sole horn lesions are more likely to occur, represents a key area for refinement. The visibility of the solar DP surface can be improved through the use of sealing materials, such as a gel pad for infilling the larger defects of the sole horns when scanning the affected claws. In contrast, the DP-SS angles were generally measurable in claws with white line disease, as this condition does not typically cause such extensive defects within the sole horn, even when severe.

The present US method of scanning the dorsal and plantar claw parts separately followed by calculating the DP-SS angle could generate slight measurement errors, resulting in slightly lower CCCs compared with the CT measurements. Visualizing point S1 together with S2 on the same dorsal US image was extremely difficult owing to the smaller field of view [11,23], resulting in angle S1–S2 being measured as larger. This limitation could be improved by utilizing a movie obtained from a moving transducer on the sole surface in the dorsal–plantar direction, showing the solar DP echotexture from the apex to the tuberculum flexorium.

Setting double standards for the DP-SS angle is required for this method to be applicable for each medial and lateral claw of a cow’s hindlimbs. Furthermore, utilizing the DP-SS angle may be limited only to the lateral claws of the hindlimbs due to negative data for the medial claws, such as the lower correlation between the value and the NBF width and less difference between CD (−) and CD (+). This limitation would be acceptable for clinical utilization, because there is an extremely high prevalence of claw horn disruption lesions in the lateral claws of hindlimbs.

### 4.4. Study Limitations

The results in the present report were obtained by using both US and CT to examine slaughterhouse specimens. They do not completely indicate the clinical applicability of the present method in the field. When compared with postmortem US examinations for bovine claws, which allow for a thorough scan, the US scanning of hindlimb claws in living animals can lead to failures in observing the solar DP surfaces and measuring the DP-SS angles. The movement of the body in the examined animals could make it difficult to obtain adequate dorsal and plantar US images, because aligning point S2 across these two US images was slightly challenging even during postmortem US scanning. The measurement values might be influenced by changes after death within bovine claws, although these were considered minimal because of the short interval between the collection and examination. Postmortem US is not completely reliable as a reference to antemortem US, because it may involve changes to the echotextures of the soft tissue layers presenting along the DP as well as the bones themselves. Thus, future clinical research on the presented method would require an investigation of the association between the DP-SS angle and other established US parameters in bovine claws, including the digital cushion (or soft tissue layer) thickness and the sole horn thickness. The digital cushion provides shock absorption to reduce the mechanical loads generated within the bovine claw when standing and walking [1,4]. The function of this structure changes depending on its quality and thickness [1,4]. The thinning of the digital cushion is simply considered to lead to a reduction in function [1,4]. Claw US helps identify a thinner digital cushion (or thinner soft tissue layers) within diseased claws, whereas thickening can occur in association with inflammatory changes [1,17,18]. A thinner digital cushion may result from the narrowing of the anatomical space between the internal sole horn margin and the solar DP surface associated with bone remodeling in the DP, as well as a true reduction in its thickness [17]. Additionally, changes in its quality and thickness may be one of the etiological factors of an NBF in the DP. Furthermore, the thickening of the sole horn in excess of 10 mm may contribute to reduced echogenicity and even the disappearance of the DP’s echotexture on US [9,11]. Thus, these parameters should be jointly evaluated to better understand the factors that influence the accuracy of DP-SS angle measurements. Additionally, the present report does not include macroscopic evaluations of these parameters based on the US and CT measurements. Basic research would be required to support clinical research in the future.

## 5. Conclusions

A DP-SS angle helps estimate the degree of an NBF in the DP within the lateral claws compared with the medial claws in a cow’s hindlimbs. A CT analysis enabled the identification of a high measurement accuracy for this value when using dorsal and plantar US images scanned separately, assuming the use of US in the field. The DP-SS angle can be utilized to create criteria for claws with a sole ulcer/sole crack or a double sole, as this angle differed greatly compared with CD (−). However, the grouping of CD (−) and CD (+) was performed based on the CT and macroscopic findings, without basic data on the nutritional state (such as the body condition score), frequency of routine trimming, milk productivity, reproductive performance, period after calving in examinations, and history of lameness. Specimens belonging to CD (−) did not always equal a healthy claw, because cows without claw diseases, as detected using CT, might present with lameness. Additionally, the DP-SS angle in the CD (+) group was not evaluated based on clinical data, including the lameness score, history of therapy, therapeutic effect, and recurrence in cows presenting with lameness. Thus, field research using US measurements of the DP-SS angle would clarify the association of the angle with these data. This would contribute to determining a definite standard value in healthy claws and an etiological threshold indicating claw horn disruption lesions, as well as creating criteria indicative of the prognosis.

## Figures and Tables

**Table 1:** Mean ± SD of CT-calculated and CT-measured DP-SS angle (°) in the claws of the hindlimbs of 105 cows.

Measurements	Medial Claws (n = 210)	Lateral Claws (n = 210)	Total Claws (n = 420)
Angle S1–S2	81.31 ± 4.11	80.84 ± 4.57	81.08 ± 4.36
Angle S2–S3	83.69 ± 4.95 ^a^	80.78 ± 6.04 ^b^	82.23 ± 5.72 ^c^
CT-calculated DP-SS angle	165.00 ± 4.20 ^a^	161.61 ± 5.69 ^b^	163.31 ± 5.27 ^c^
CT-measured DP-SS angle	164.52 ± 4.13 ^a^	161.21 ± 5.57 ^b^	162.86 ± 5.17 ^c^

^a–c^ Data in the same row with different superscript letters are significantly different (*p* < 0.01).

**Table 2:** Mean ± SD of DP-SS angle (°) measured using CT in claws with or without claw diseases from the hindlimbs of 242 cows.

Specimens	Medial Claws		Lateral Claws		Total Claws	
	n	DP-SS Angle	n	DP-SS Angle	n	DP-SS Angle
CD (+)	86	163.77 ± 4.50 ^a^	159	158.55 ± 6.46 ^b,d,f^	245	160.38 ± 6.36 ^b,d,f^
Double sole	28	162.30 ± 5.14	36	158.21 ± 6.31 ^h^	64	160.00 ± 6.17 ^f,h^
White line disease	18	165.38 ± 4.32	35	162.27 ± 4.97 ^f^	53	163.33 ± 4.98 ^f^
Sole ulcer/sole crack	3	164.15 ± 2.42 ^a^	47	154.38 ± 4.94 ^b,g,h^	50	154.97 ± 5.36 ^g,h^
Interdigital dermatitis	10	163.45 ± 3.65	7	160.72 ± 6.14	17	162.32 ± 5.02 ^f^
Thin sole/thin sole toe ulcer	21	164.32 ± 4.26	16	163.47 ± 5.03 ^f^	37	163.96 ± 4.63 ^f^
Others	6	164.16 ± 1.63	18	157.66 ± 6.63	24	159.29 ± 6.44 ^h^
CD (−)	398	164.03 ± 3.82 ^a^	325	161.63 ± 4.17 ^b,e,i^	723	162.95 ± 4.16 ^c,e,i^
Total	484	163.98 ± 3.95 ^a^	484	160.63 ± 5.24 ^b,e^	968	162.30 ± 4.94 ^c,e^

^a–c^ Data in the same row with different superscript letters are significantly different (*p* < 0.01). ^d,e^ Data in the same column [CD (+) vs. CD (−) vs. total groups] with different superscript letters are significantly different (*p* < 0.01). ^f,g^ Data in the same column [six types of claw disease vs. CD (+)] with different superscript letters are significantly different (*p* < 0.01). ^h,i^ Data in the same column [six types of claw disease vs. CD (−)] with different superscript letters are significantly different (*p* < 0.01).

**Table 3:** Mean ± SD of the NBF width (mm) measured using CT in claws with or without claw diseases from the hindlimbs of 242 cows.

Specimens	Medial Claws		Lateral Claws		Total Claws	
	n	NBF Width	n	NBF Width	n	NBF Width
CD (+)	86	1.76 ± 1.06 ^a,d^	159	3.49 ± 2.44 ^b,d,g^	245	2.88 ± 2.22 ^b,d,g^
Double sole	28	1.87 ± 1.32 ^a^	36	3.65 ± 2.56 ^b,i^	64	2.87 ± 2.29 ^g,i^
White line disease	18	1.44 ± 0.77	35	2.36 ± 2.06 ^g^	53	2.05 ± 1.79 ^g^
Sole ulcer/sole crack	3	2.61 ± 0.95 ^a^	47	5.05 ± 2.17 ^h,i^	50	4.90 ± 2.20 ^h,i^
Interdigital dermatitis	10	1.55 ± 0.85	7	2.40 ± 1.67	17	1.90 ± 1.32 ^g^
Thin sole/thin sole toe ulcer	21	1.87 ± 0.97	16	1.86 ± 1.01 ^g^	37	1.86 ± 0.99 ^g^
Others	6	1.84 ± 0.62	18	3.19 ± 2.30	24	2.85 ± 2.09 ^g,i^
CD (−)	398	1.40 ± 0.82 ^a,e^	325	1.99 ± 1.44 ^b,e,j^	723	1.66 ± 1.18 ^c,e,j^
Total	484	1.46 ± 0.88 ^a,e^	484	2.48 ± 1.96 ^b,f^	968	1.97 ± 1.60 ^c,f^

^a–c^ Data in the same row with different superscript letters are significantly different (*p* < 0.01). ^d–f^ Data in the same column [CD (+) vs. CD (−) vs. total groups] with different superscript letters are significantly different (*p* < 0.01). ^g,h^ Data in the same column [six types of claw diseases vs. CD(+)] with different superscript letters are significantly different (*p* < 0.01). ^i,j^ Data in the same column [six types of claw diseases vs. CD (−)] with different superscript letters are significantly different (*p* < 0.01).

**Table 4:** Rates (%) of measuring DP-SS angles of <150°, 150–<160°, and ≥160° using CT in 6 types of claw diseases and CD (−) group in hindlimbs of 242 cows.

Specimens	Medial Claws				Lateral Claws				Total Claws			
	n	<150°	150–<160°	≥160°	n	<150°	150–<160°	≥160°	n	<150°	150–<160°	≥160°
Double sole	28	3.6 ^a^	21.4	75.0	36	11.1 ^a^	47.2 ^a^	41.7 ^a^	64	7.8 ^a^	35.9 ^a^	56.3 ^a,c^
White line disease	18	0.0	5.6	94.4	35	2.9 ^b,c^	25.7 ^a^	71.4 ^a^	53	1.9 ^b,c^	18.9 ^a^	79.2 ^c^
Sole ulcer/sole crack	3	0.0	0.0	100.0	47	12.8 ^d,e^	80.9 ^b^	6.4 ^b^	50	12.0 ^d,e^	76.0 ^b^	12.0 ^d^
Interdigital dermatitis	10	0.0	10.0	90.0	7	0.0	28.6	71.4 ^a^	17	0.0	17.6 ^a^	82.4 ^c^
Thin sole/thin sole toe ulcer	21	0.0	14.3	85.7	16	0.0 ^g^	18.8 ^a^	81.3 ^a^	37	0.0 ^f,g^	16.2 ^a^	83.8 ^c^
Others	6	0.0	0.0	100.0	18	11.1 ^d,h,i^	44.4	44.4 ^a^	24	8.3 ^d,h,i^	33.3 ^a^	58.3 ^c^
CD (−)	398	0.0 ^b^	13.8	86.2	325	0.6 ^b,f,j^	33.2 ^a^	66.2 ^a^	723	0.3 ^b,f,j^	22.5 ^a^	77.2 ^b,c^
Total	484	0.2	13.6	86.2	484	3.1	38.2	58.7	968	1.7	25.9	72.4

^a–j^ Data in the same column with different superscript letters are significantly different between the 6 types of claw diseases and CD (−) group (*p* < 0.01).

**Table 5:** Rates (%) of measuring DP-SS angles using US for 6 types of claw diseases and CD (−) group in cow hindlimbs.

Specimens	Number of Examined Claws	Number of Measured Claws	Measurement Rate	DP-SS Angle (°)
Double sole	35	17	48.6 ^a^	159.96 ± 6.53
White line disease	4	4	100.0	164.13 ± 2.06
Sole ulcer/sole crack	30	21	70.0 ^a^	156.73 ± 6.58 ^a^
Interdigital dermatitis	4	4	100.0	163.80 ± 3.11
Thin sole/thin sole toe ulcer	5	5	100.0	159.99 ± 4.00
Others	9	8	88.9	159.47 ± 4.99
CD (−)	461	409	88.7 ^b^	163.03 ± 4.52 ^b^
Total	548	468	85.4	162.55 ± 4.93

^a,b^ Data in the same column with different superscript letters are significantly different (*p* < 0.01).

## Data Availability

The original contributions presented in this study are included in the article. Further inquiries can be directed to the corresponding author.

## Abbreviations

The following abbreviations are used in this manuscript:

ANOVA	One-way analysis of variance
CCC	Concordance correlation coefficient
CT	Computed tomography
CD (+)	Claws in which claw diseases were detected
CD (−)	Claws in which claw diseases were not detected
DP	Distal phalanx
NBF	New bone formation
SD	Standard deviation
SS	Solar surface
S1	A point at the most apex margin of the distal phalanx
S2	A point at the deepest concavity of the solar surface of the distal phalanx
S3	A point at the tuberculum flexorium
US	Ultrasonography
